# Supplementary material for: Real-time spatiotemporal optimization during imaging
Source: Commun Eng. 2025 Mar 31;4:61. doi: 10.1038/s44172-025-00391-9 (PMC11958730; doi:10.1038/s44172-025-00391-9)
Supplement: Supplementary file 1 — Description of Additional Supplementary Files [file 44172_2025_391_MOESM1_ESM.pdf]

Description of Additional Supplementary Files

File name: Supplementary Data 1

Description: Animated GIFs looking at 4D tomographs through the middle of the ITV.

The ITV contour is shown in yellow, spine canal contour in blue (these contours are gnerally used by clinicians for patient alignment).

Note that the contours are directly from the planning CT and the patients have not neccesarily been aligned.

Each case is supposed to be representative of the study cohort, plus the worst STO200 image as quantified by CNR.
