# Supplementary figures and images for: Real-time spatiotemporal optimization during imaging
Source: Commun Eng. 2025 Mar 31;4:61. doi: 10.1038/s44172-025-00391-9 (PMC11958730; doi:10.1038/s44172-025-00391-9)

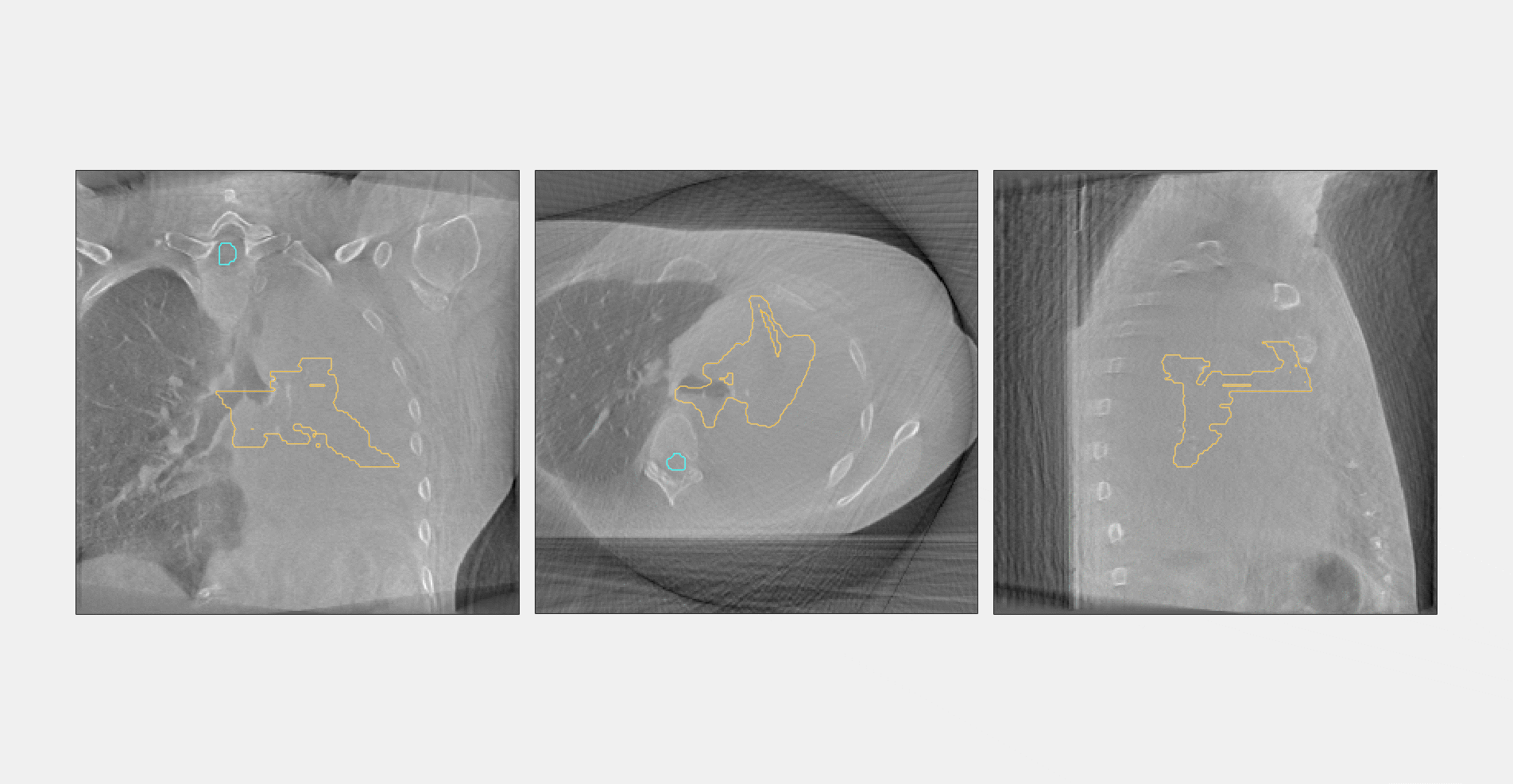

Supplement: Supplementary file 4 — Supplementary data [file 44172_2025_391_MOESM4_ESM.zip › supplementary/Case 1/conv.gif]

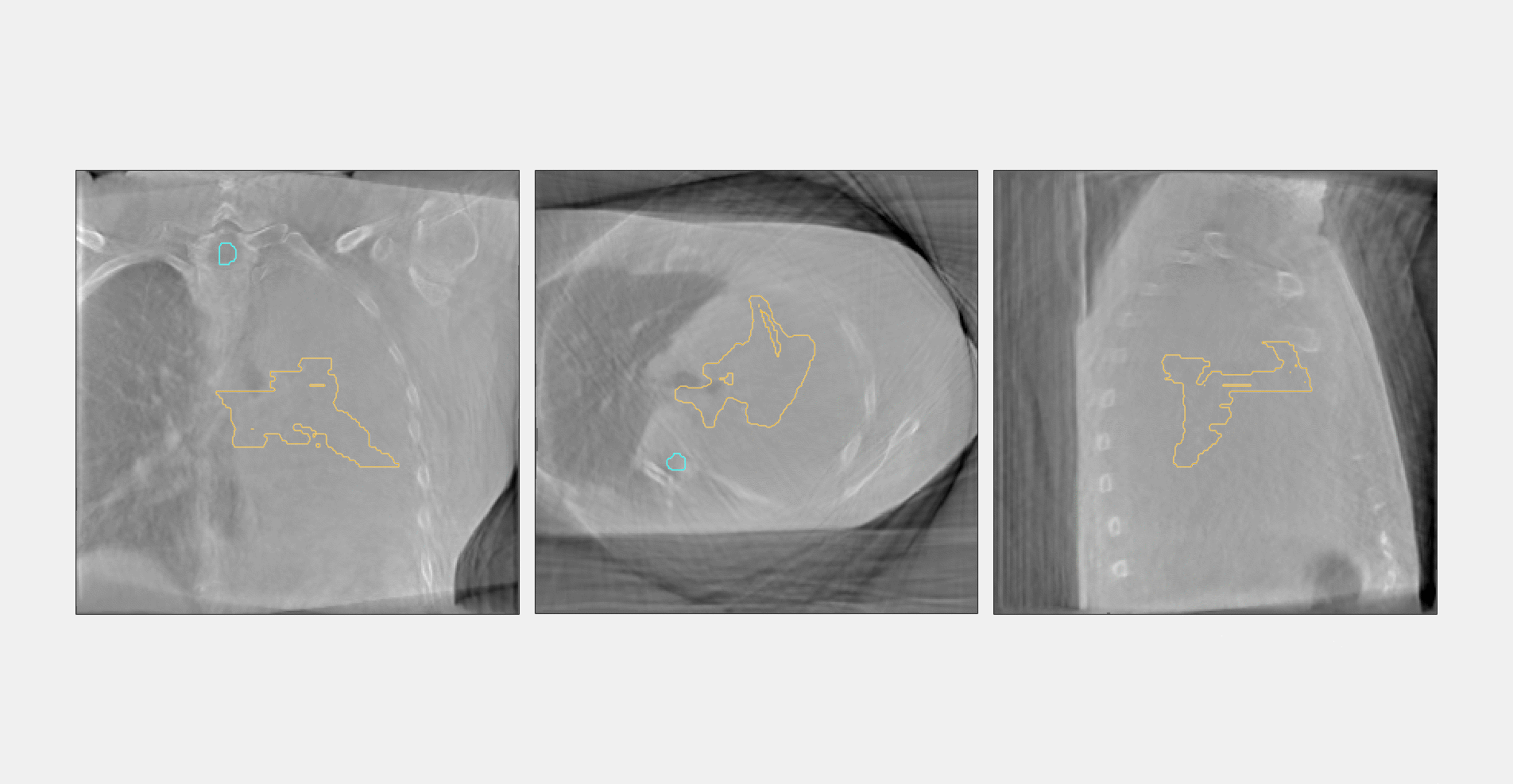

Supplement: Supplementary file 4 — Supplementary data [file 44172_2025_391_MOESM4_ESM.zip › supplementary/Case 1/STO200.gif]

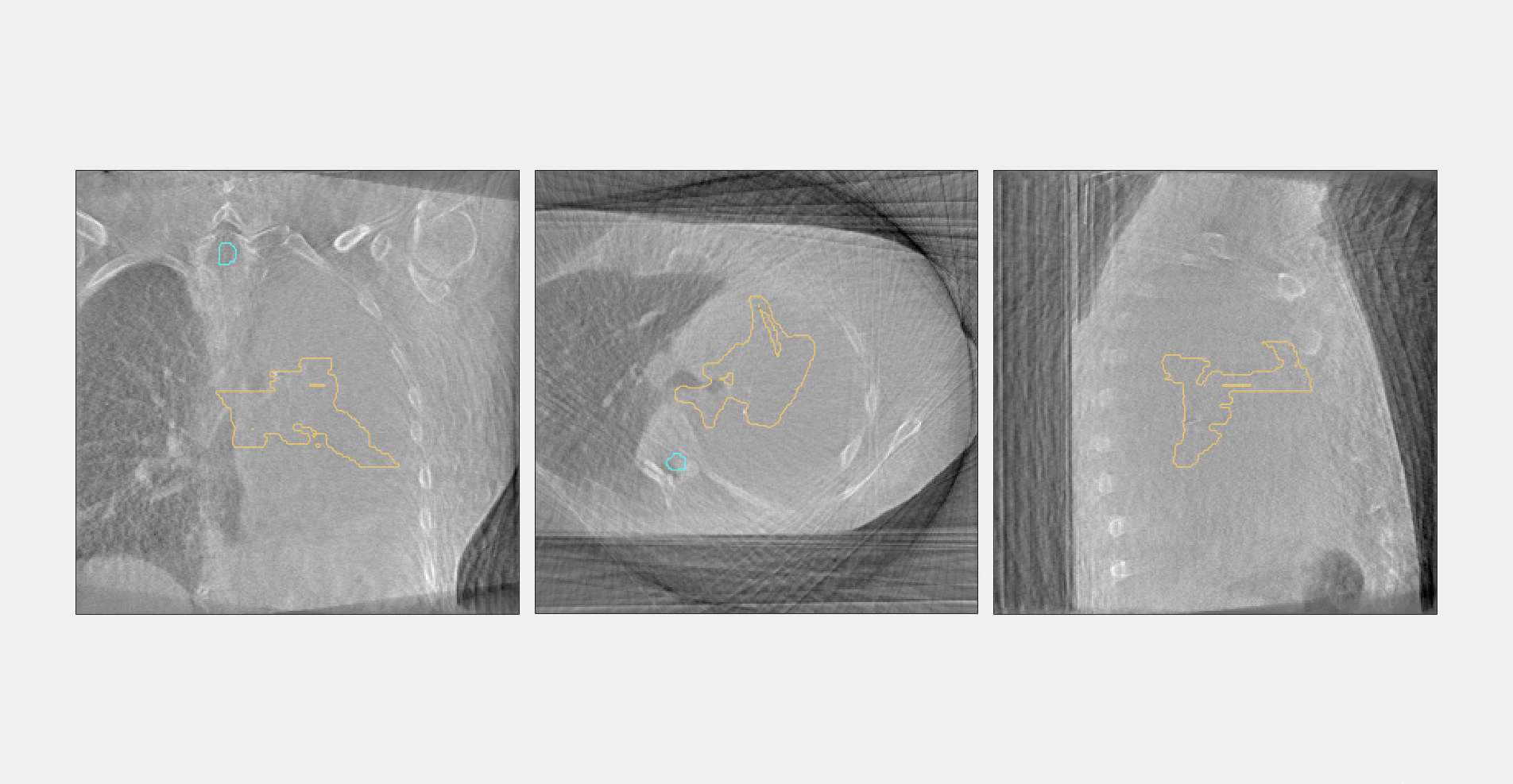

Supplement: Supplementary file 4 — Supplementary data [file 44172_2025_391_MOESM4_ESM.zip › supplementary/Case 1/STO600.gif]

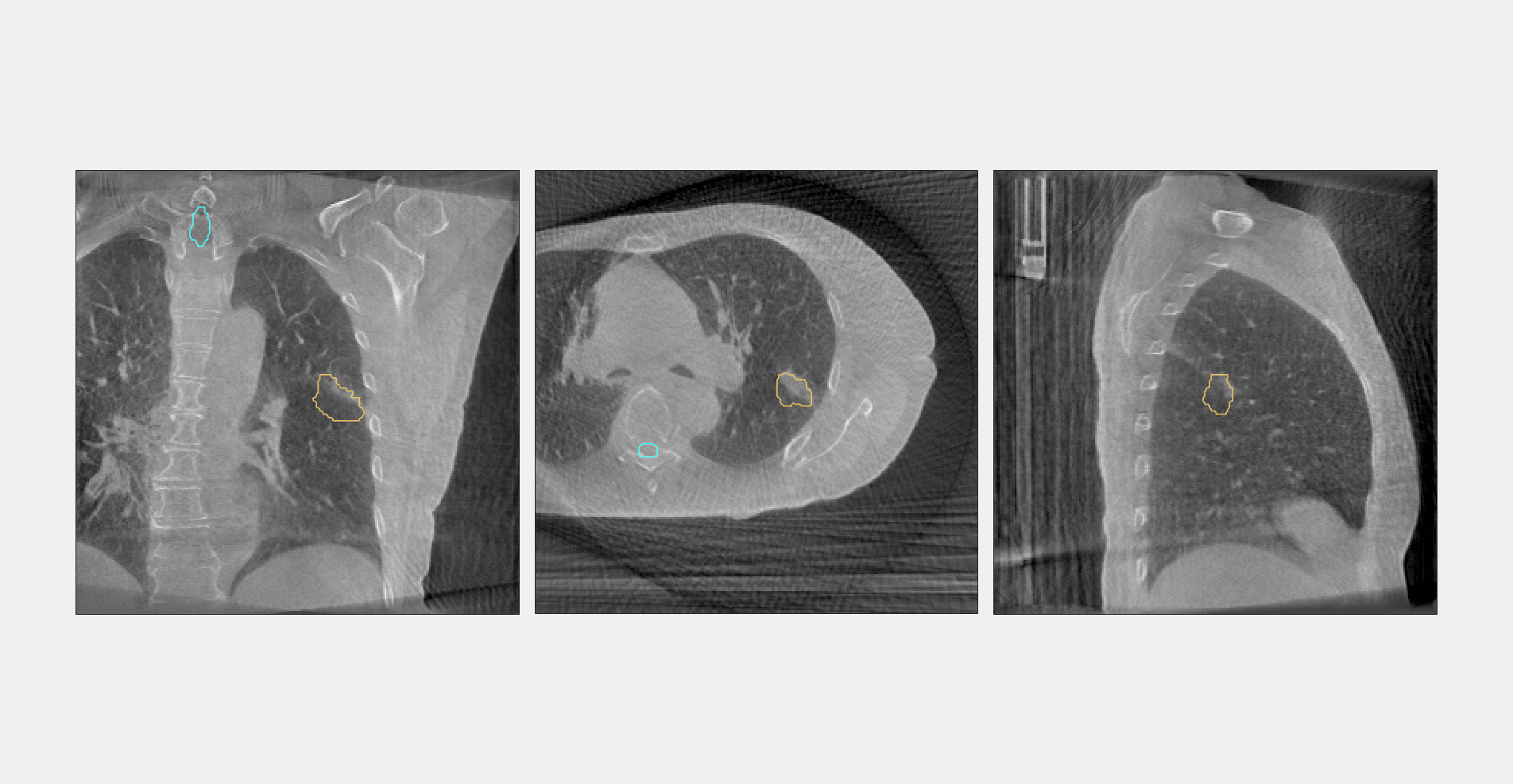

Supplement: Supplementary file 4 — Supplementary data [file 44172_2025_391_MOESM4_ESM.zip › supplementary/Case 2/conv.gif]

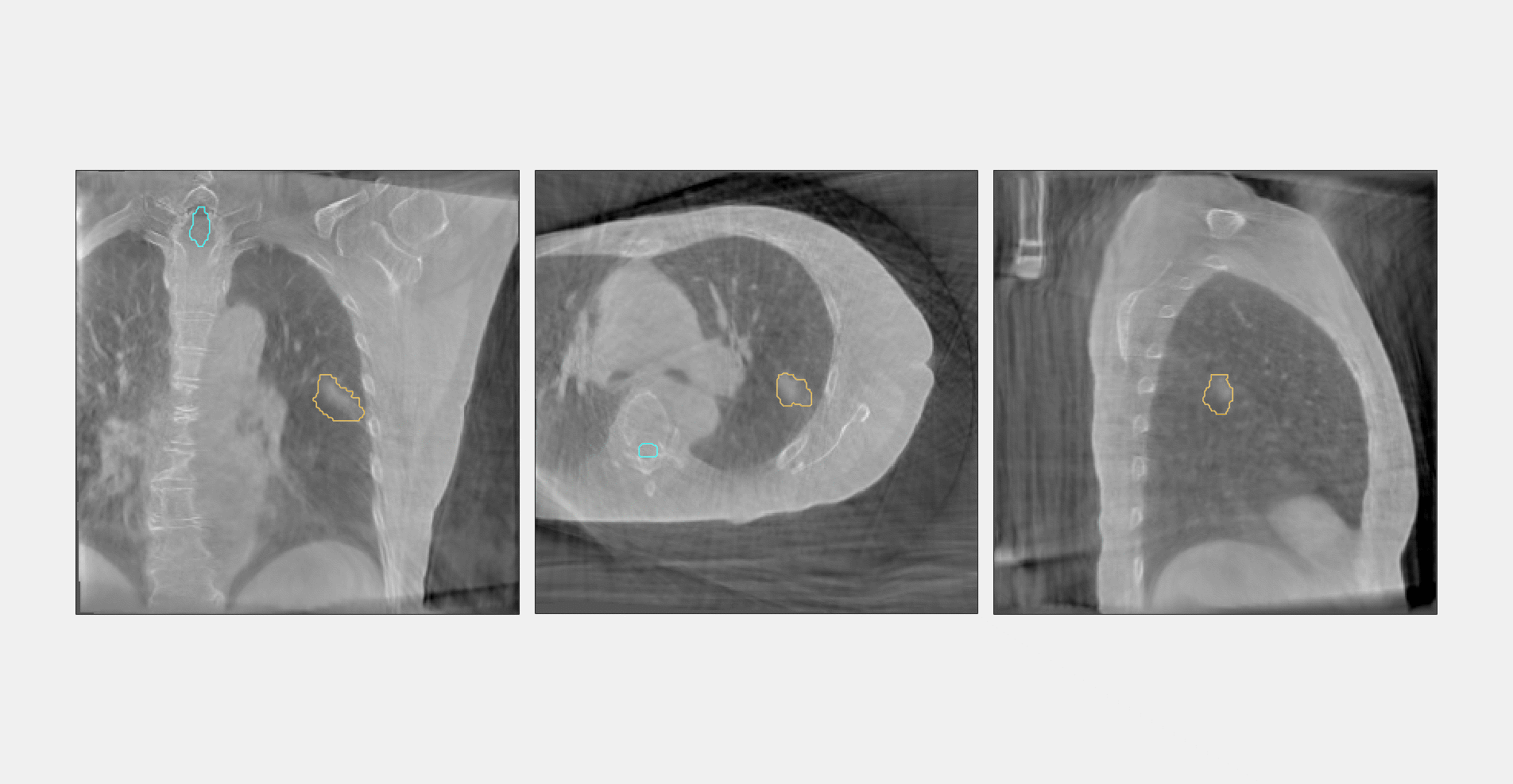

Supplement: Supplementary file 4 — Supplementary data [file 44172_2025_391_MOESM4_ESM.zip › supplementary/Case 2/STO200.gif]

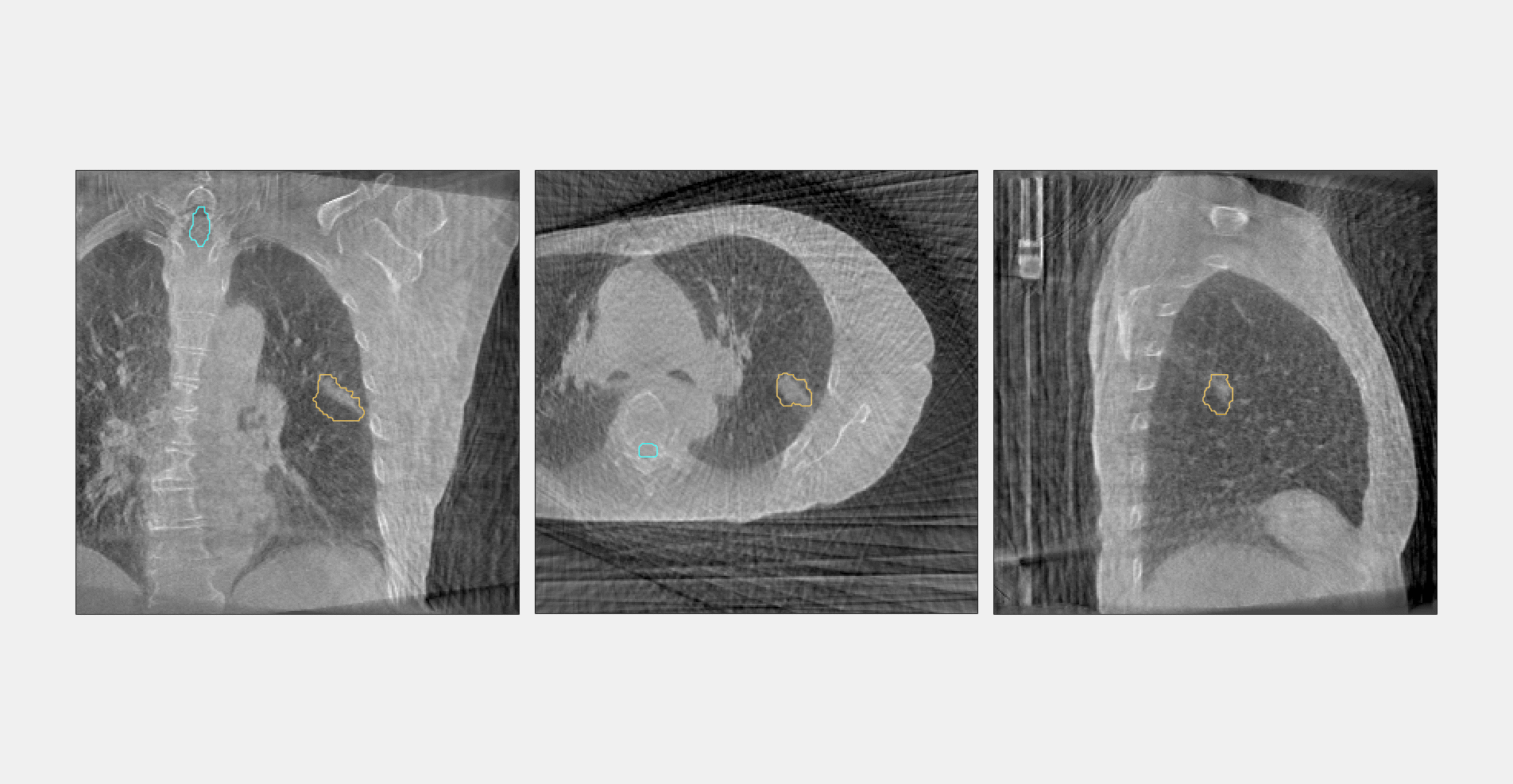

Supplement: Supplementary file 4 — Supplementary data [file 44172_2025_391_MOESM4_ESM.zip › supplementary/Case 2/STO600.gif]

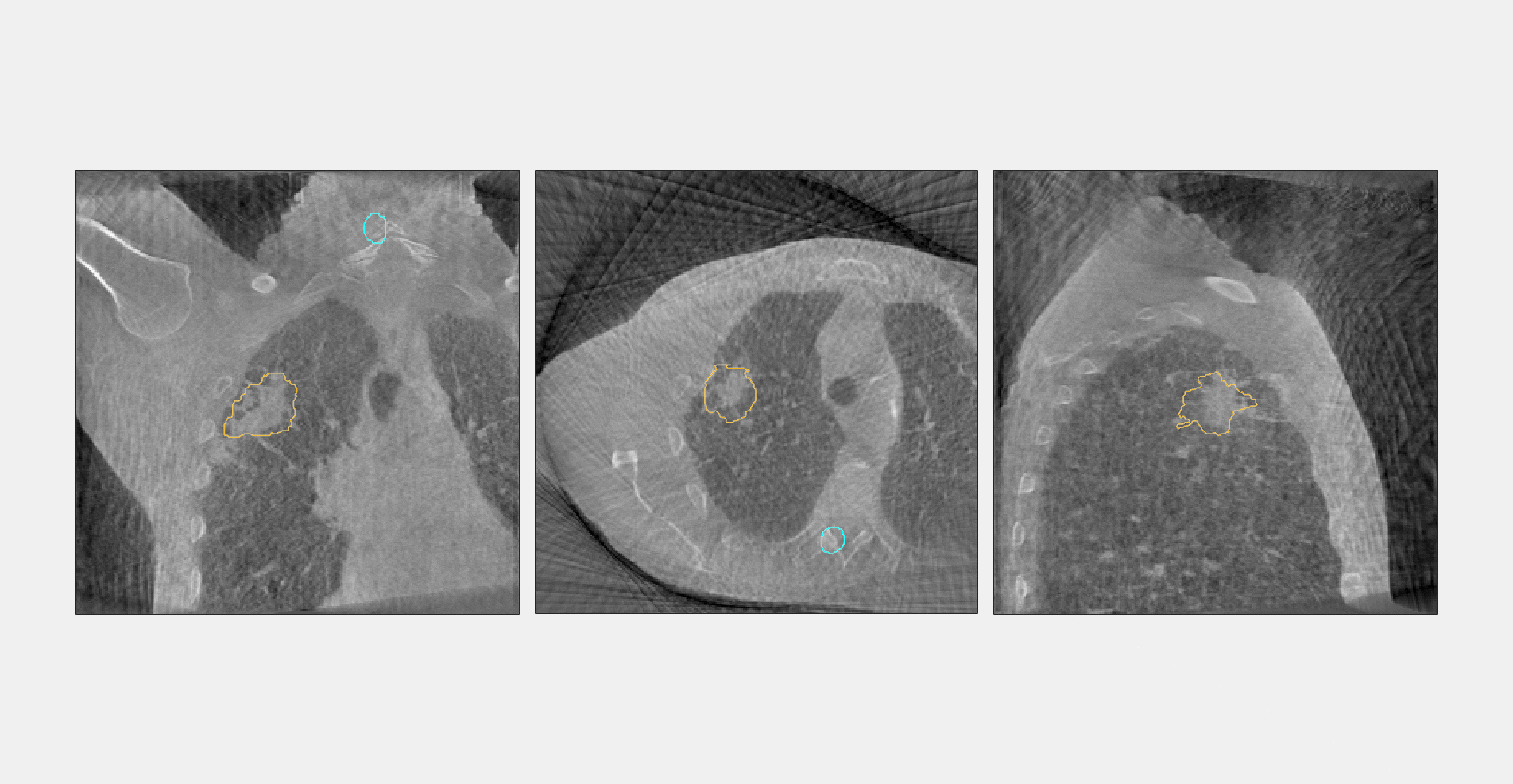

Supplement: Supplementary file 4 — Supplementary data [file 44172_2025_391_MOESM4_ESM.zip › supplementary/Case 3/conv.gif]

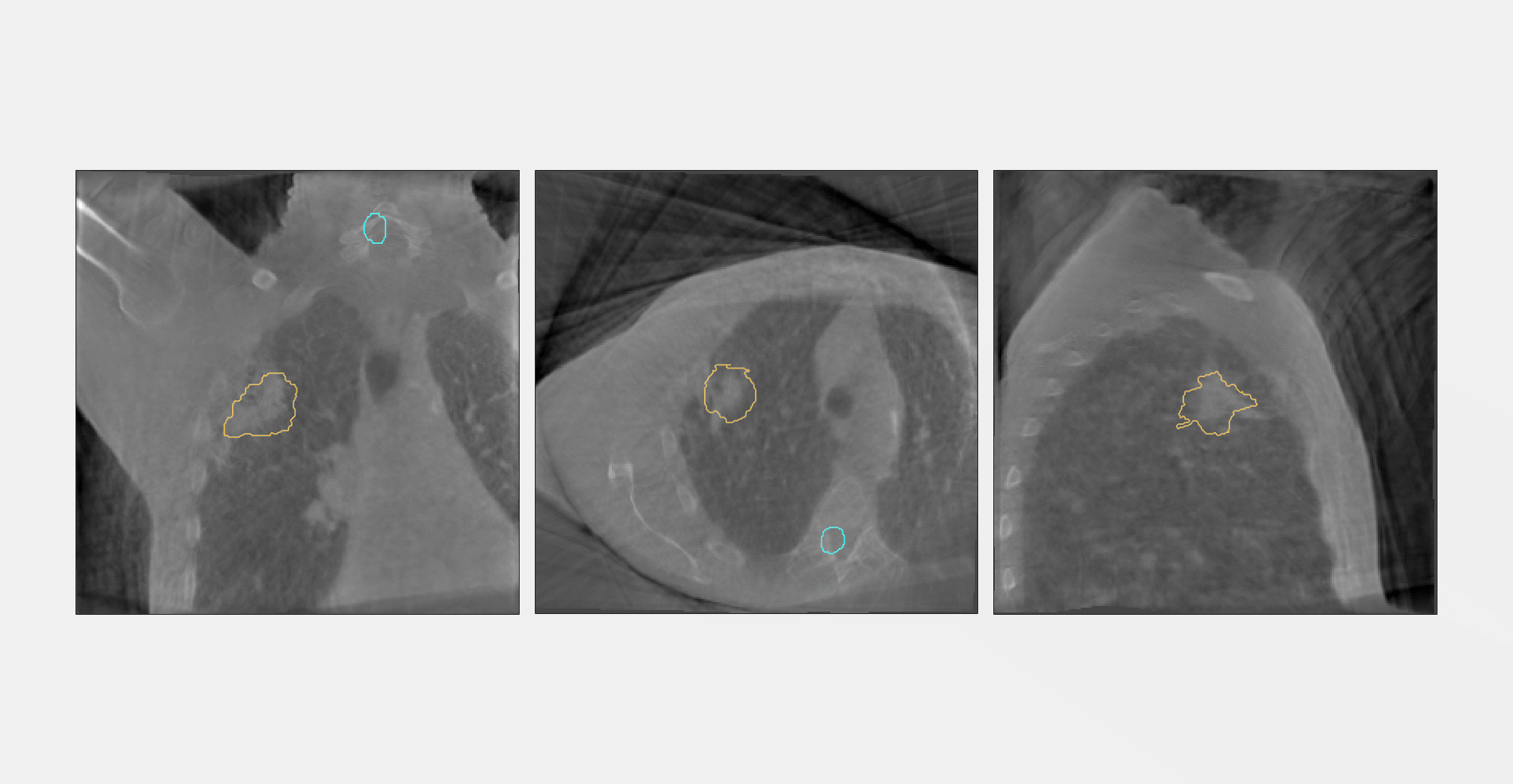

Supplement: Supplementary file 4 — Supplementary data [file 44172_2025_391_MOESM4_ESM.zip › supplementary/Case 3/STO200.gif]

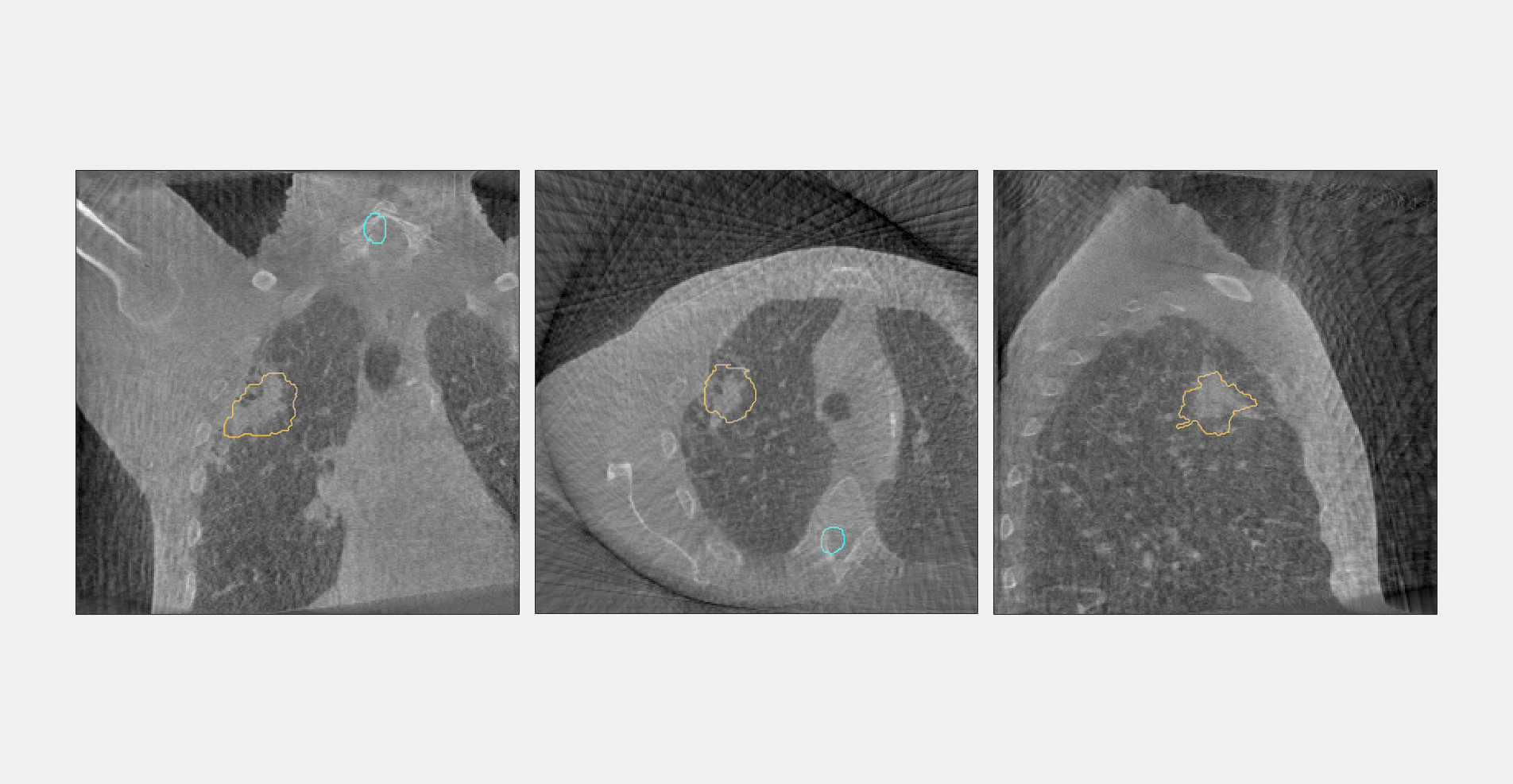

Supplement: Supplementary file 4 — Supplementary data [file 44172_2025_391_MOESM4_ESM.zip › supplementary/Case 3/STO600.gif]

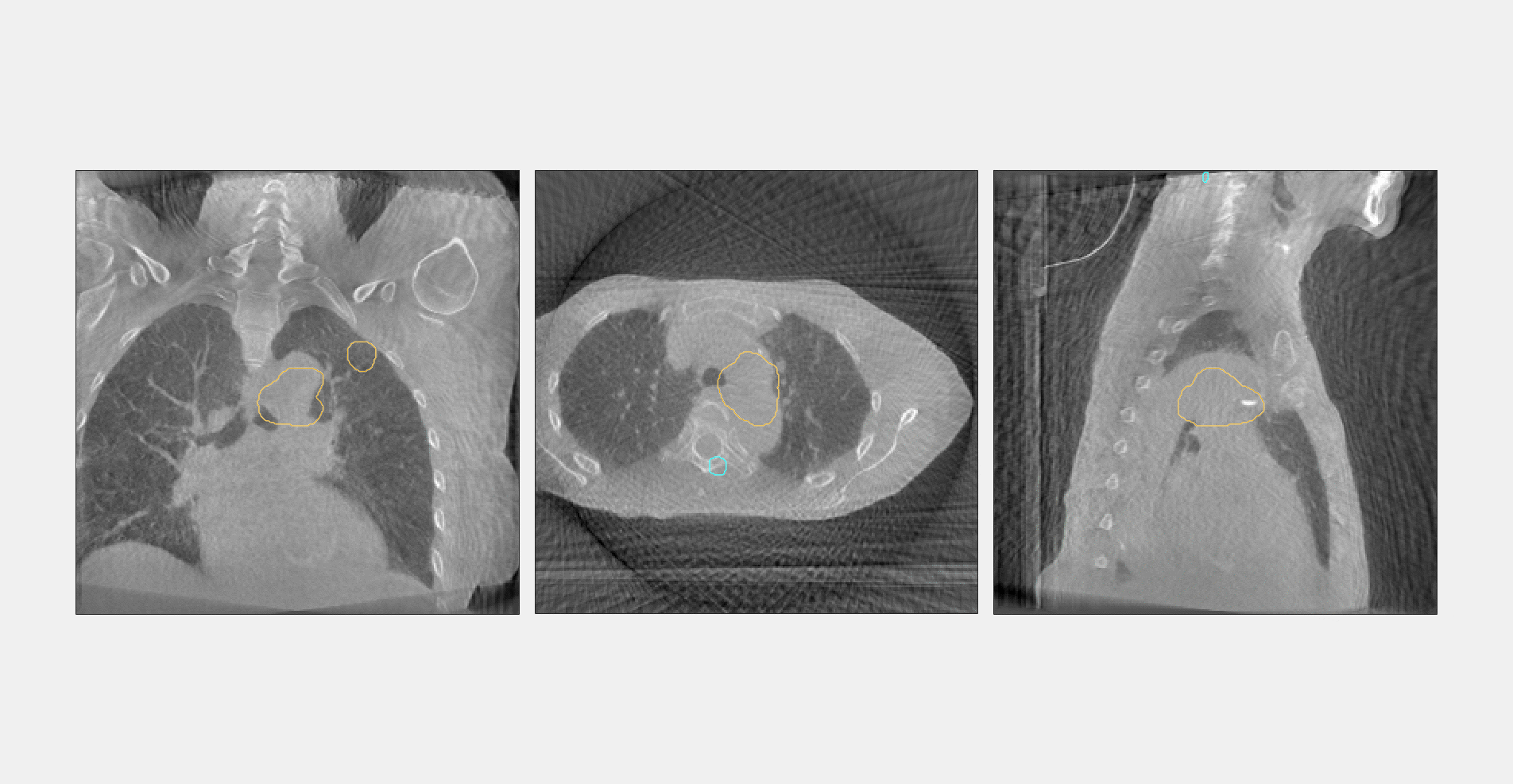

Supplement: Supplementary file 4 — Supplementary data [file 44172_2025_391_MOESM4_ESM.zip › supplementary/Case 4/conv.gif]

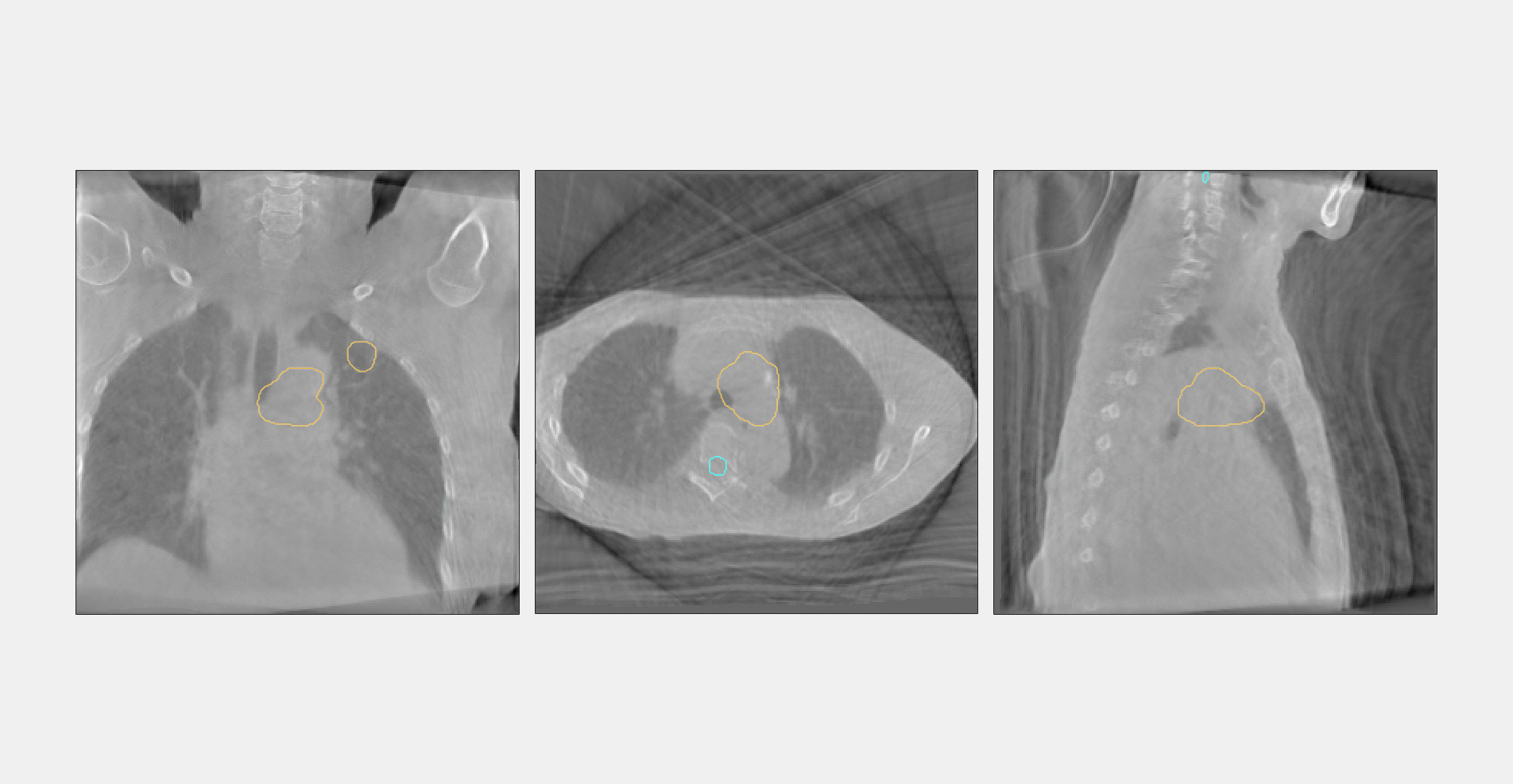

Supplement: Supplementary file 4 — Supplementary data [file 44172_2025_391_MOESM4_ESM.zip › supplementary/Case 4/STO200.gif]

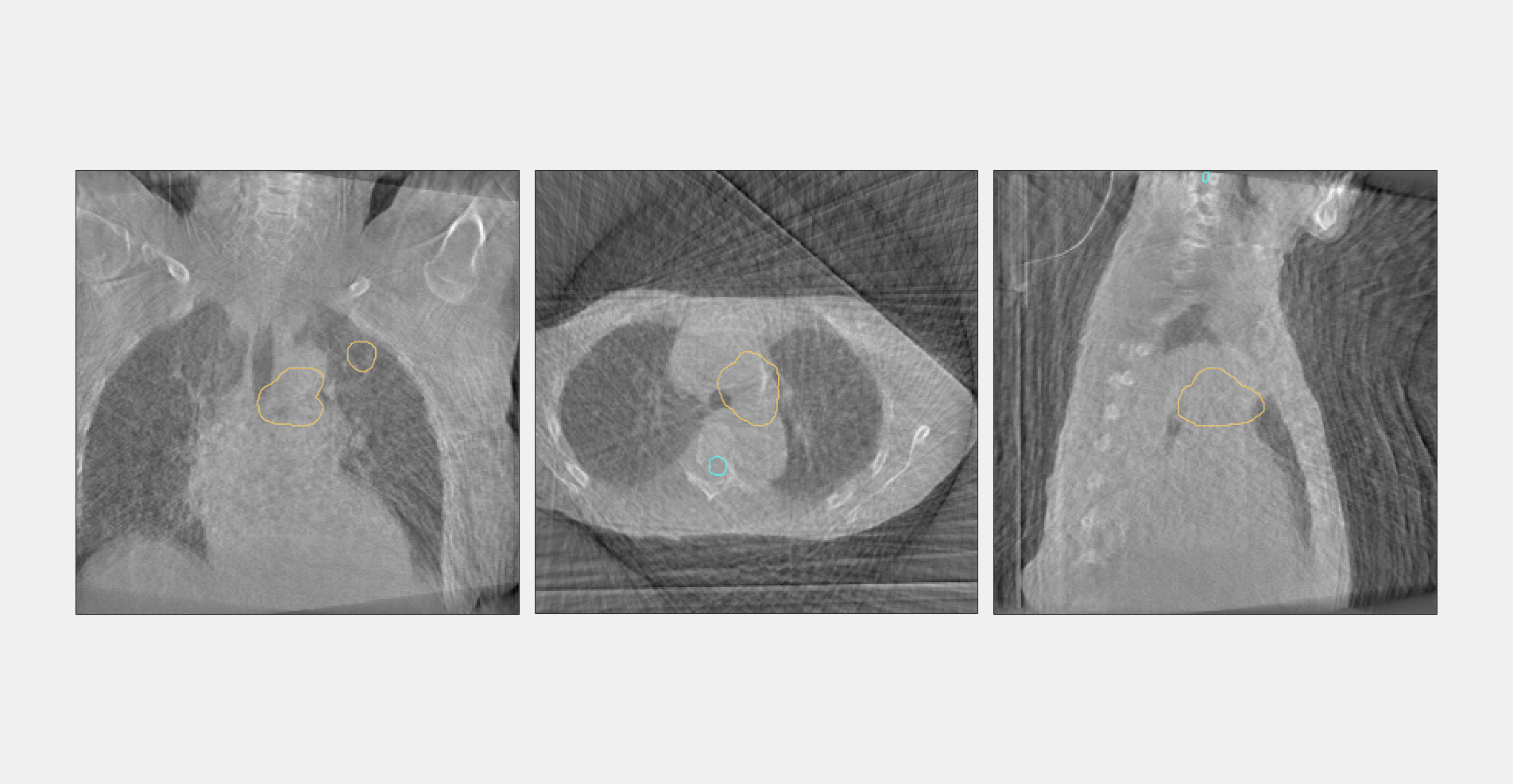

Supplement: Supplementary file 4 — Supplementary data [file 44172_2025_391_MOESM4_ESM.zip › supplementary/Case 4/STO600.gif]

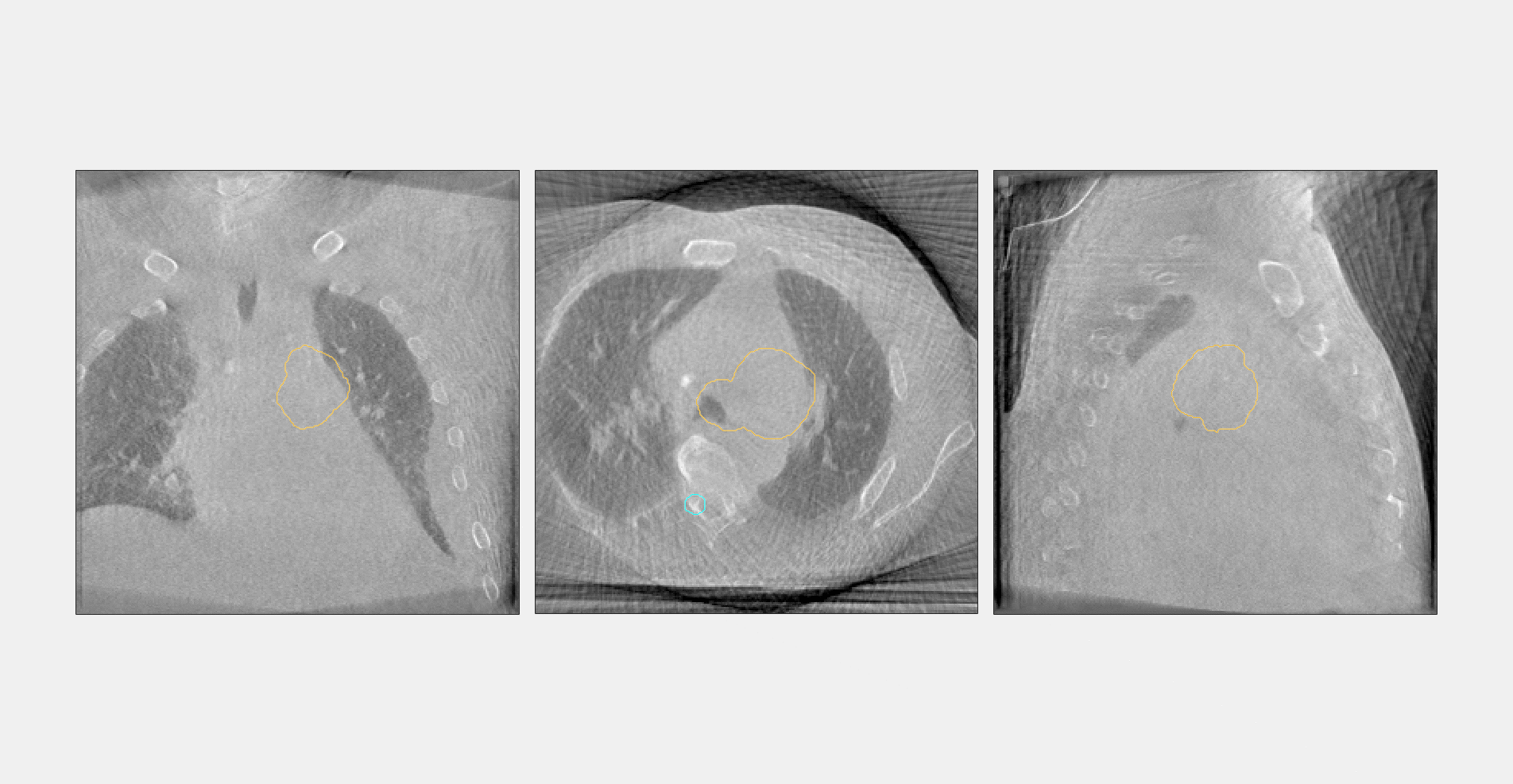

Supplement: Supplementary file 4 — Supplementary data [file 44172_2025_391_MOESM4_ESM.zip › supplementary/Case 5 - worst CNR/conv.gif]

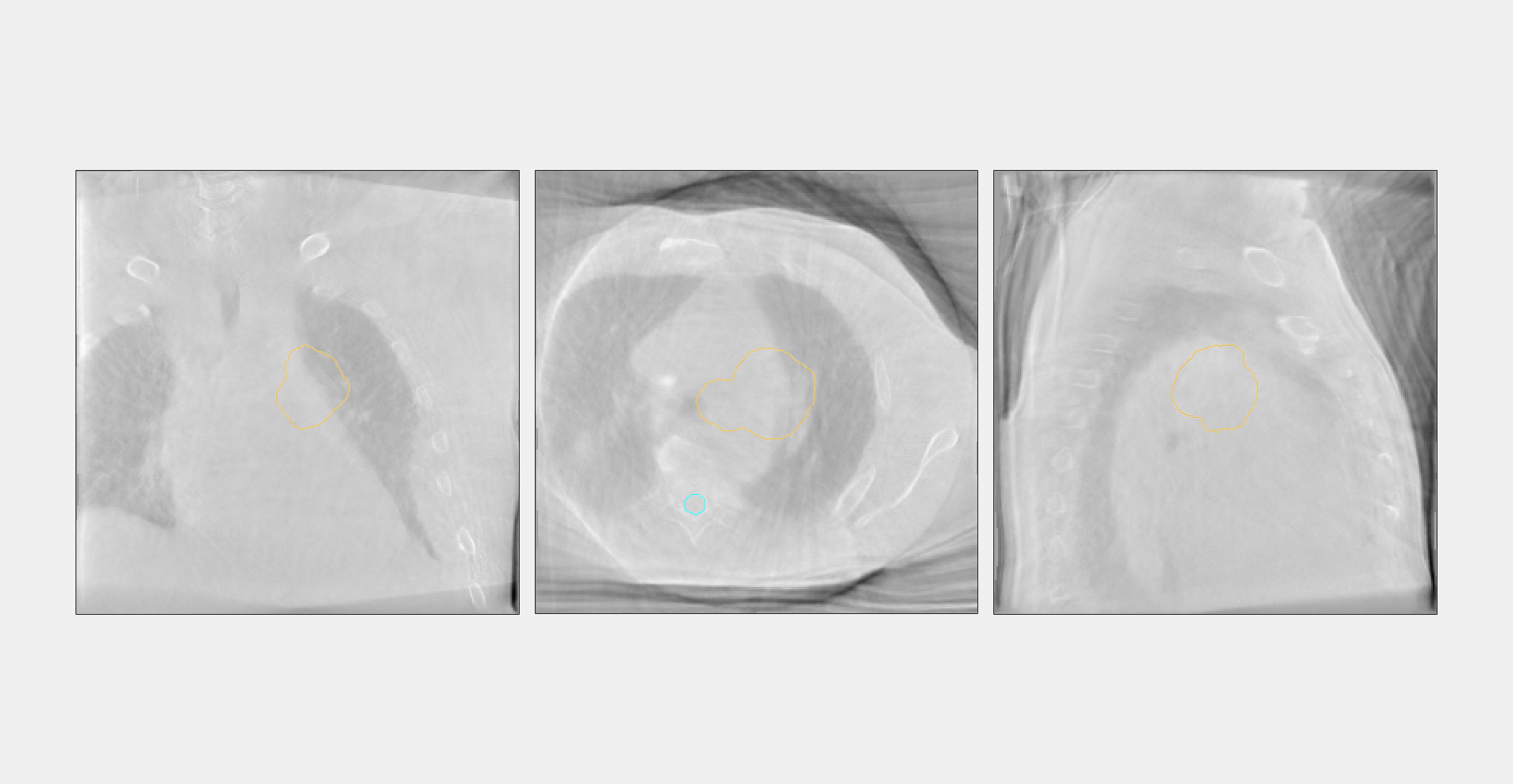

Supplement: Supplementary file 4 — Supplementary data [file 44172_2025_391_MOESM4_ESM.zip › supplementary/Case 5 - worst CNR/STO200.gif]

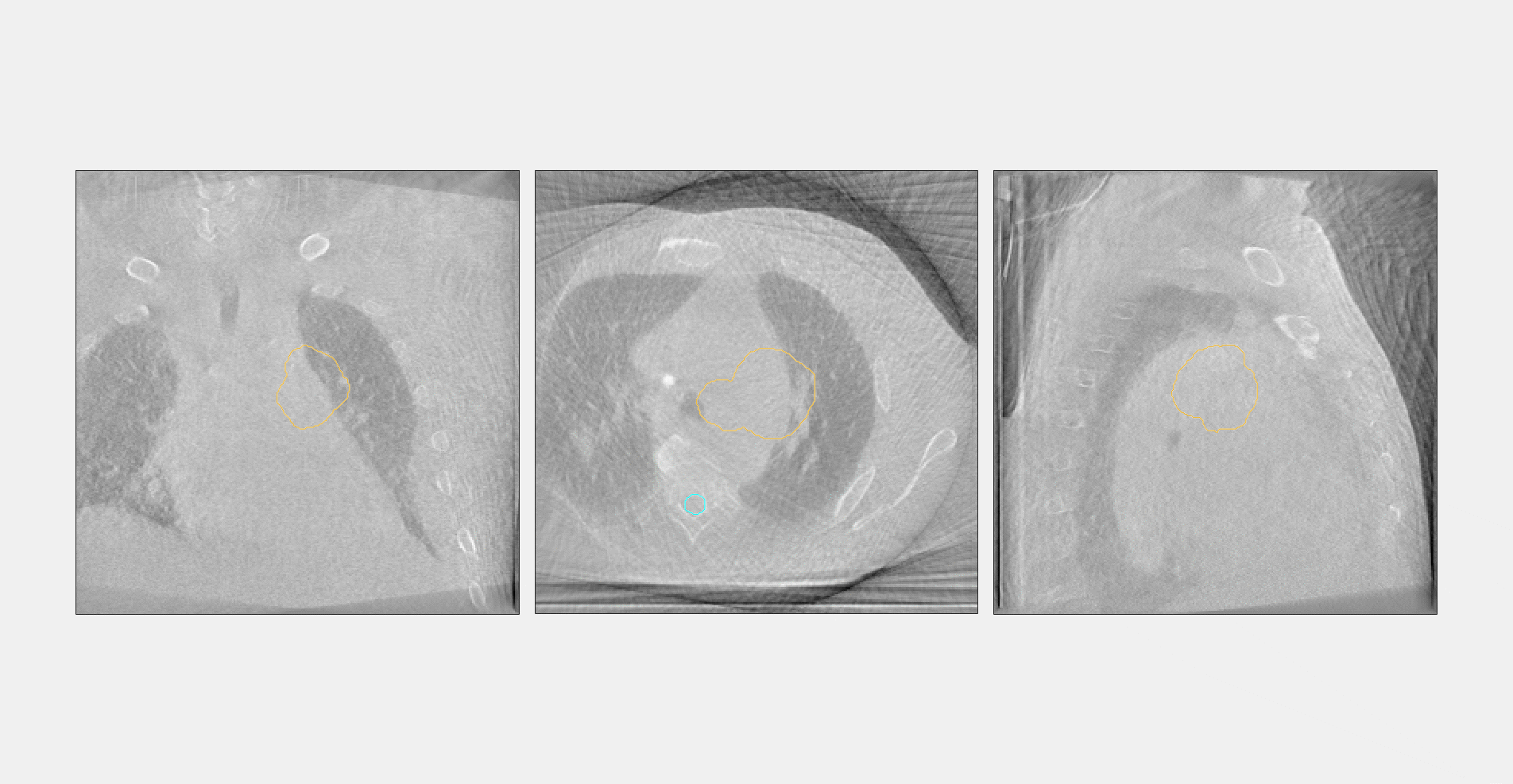

Supplement: Supplementary file 4 — Supplementary data [file 44172_2025_391_MOESM4_ESM.zip › supplementary/Case 5 - worst CNR/STO600.gif]
